# H1N1-Associated Encephalitis in a Child with Acute Myeloblastic Leukemia and Bacteremia due to Klebsiella Pneumoniae

**DOI:** 10.5505/tjh.2012.80947

**Published:** 2012-03-05

**Authors:** Lale Olcay, Seda Öztürkmen, Zafer Bıçakçı, Arzu Akyay, Gülşen İskender, Kamuran Türker Sayılır, Nil Çetin, Saadet Dikmen Menteş, Özlem Kapusuz, Mehmet Ertem

**Affiliations:** 1 Dr A.Y. Ankara Oncology Training and Research Hospital, Department of Pediatric Hematology, Ankara, Turkey; 2 Dr A.Y. Ankara Oncology Training and Research Hospital, Department of Infectious Diseases, Ankara, Turkey; 3 Dr A.Y. Ankara Oncology Training and Research Hospital, Department of Neurology, Ankara, Turkey; 4 Dr A.Y. Ankara Oncology Training and Research Hospital, Department of Anesthesia, Ankara, Turkey; 5 Ankara University, School of Medicine, Department of Pediatrics, Division of Pediatric Hematology, Ankara, Turkey

**Keywords:** H1N1, Encephalitis, Acute myeloblastic leukemia

## Abstract

Herein we present a neutropenic 16-year-old female with acute myeloblastic leukemia that developed recurrentgeneralized seizures while receiving antimicrobial therapy (including oseltamivir) due to pneumonia, bacteremiaof Klebsiella pneumoniae, and H1N1 infection. The patient’s seizures were controlled using assisted ventilation.Electroencephalography showed that the patient had encephalopathy. Cranial computed tomography (CT), magneticresonance imaging (MRI), and MRI angiography findings were normal. The patient fully recovered without sequelae.This case indicates that during pandemics of influenza-like diseases H1N1 infection should always be a consideration.

## Abstract (tr)

Burada, pnömoni, Klebsiella pneumonia bakteriyemisi ve H1N1 enfeksiyonu nedenleri ile antimikrobiyal tedavi (oseltamivir dahil) almakta iken tekrarlayıcıç jeneralize nöbetler geçiren ve nötropenik olan, 16 yaşında, akut miyeloblastik lösemili bir kız olguyu sunmaktayız. Hastanın nöbetleri, yardımlı ventilasyon ile kontrol edilebildi. Elektroensefalografi ensefalopati ile uyumlu olarak saptandı. Bilgisayarlı beyin tomografisi, magnetik rezonans görüntüleme (MRG), MRG anjiografi normal bulundu. Hasta sekelsiz, tamamen iyileşti. Bu olgu, influenza-benzeri hastalıkların pandemileri sırasında H1N1’in daima akılda tutulması gerektiğini vurgulamaktadır.

## INTRODUCTION

On 11 June 2009 the World Health Organization(WHO) announced the first pandemic of the 21st century,which was caused by a novel influenza A virus (H1N1).Immunosuppressed individuals and those with hematologicaldisorders have a high risk of H1N1-associatedcomplications [1]. Herein we present a patient with leukemiaand H1N1 infection that developed encephalitis whilehaving febrile neutropenia and bacteremia due to Klebsiellapneumoniae.

## CASE REPORTS

A 16-year-old female with acute myeloblastic leukemia (M4) was started the AML -BFM-2004 chemotherapy protocol on October 02, 2009, which resulted in bone marrow remission on the 15th day of therapy. After receiving the second induction therapy (November 10-13, 2009), she developed febrile neutropenia (November 21, 2009). At that time her physical examination was normal, other than partial alopecia, nasal discharge, and excessive tearing. Chest X-ray was normal and occipitomental (Waters’ view) X-ray was consistent with sinusitis. After taking blood, urine, oropharynx and stool cultures, cefoperazone-sulbactam and amikacin were started; prophylactic trimethoprim-sulfamethoxazole was continued (5 mg/kg of trimethoprim). 72 h later the patient’s fever persisted and teicoplanin was added to the treatment. Based on the suspicion of 2009 H1N1 infection, a nasopharyngeal swab specimen was obtained, oseltamivir (2x75 mg d^–1^) was added empirically, and the patient was quarantined. No bacteria grew in urine culture, oropharynx culture revealed normal throat flora, and stool culture was negative for Salmonella shigella species. Blood culture was positive for Klebsiella pneumoniae ESBL, which was sensitive to imipenem; therefore, cefoperazone-sulbactam was withdrawn and imipenem was administered. On the sixth day of fever crepitant rales were heard in the patient’s basal lung fields. High-resolution computed tomography (HRCT) showed infiltration of scattered nodular lesions in the bilateral basal and right medium lung fields. Galactomannan was negative. The dosage of trimethoprim- sulfamethoxazole was increased (20 mg kg^–1^ of trimethoprim) and clarithromycin was added to the treatment regimen. On November 28, 2009, the patient had a generalized tonic-clonic seizure that lasted nearly 3 min, after which cardiopulmonary arrest developed, when she was on imipenem, oseltamivir, amikacin, teicoplanin, tritrimethoprim-sulfamethoxazole (high dose), clarithromycin therapies, for 3,3,7,4,1,1 days respectively.

Cardiopulmonary resuscitation was implemented. She then had consecutive generalized tonic-clonic seizures that could not be controlled using midazolam or phenytoin; therefore, she was intubated; thiopentone sodium was administered under assisted ventilation and the patient was deeply sedated with propofol (2 mg/kg/hour) in Intensive Care Unit (ICU).

On November 28, 2009, her Hb was 126 g L^–1^, WBC was 0.9 x 10^9^ L^–1^, absolute neutrophil count (ANC) was 0.06 x 10^9^ mm–3, and PLT count was 27.7 x 10^9^ L–^1^. Biochemistry (glucose, sodium, potassium, BUN, creatinine, uric acid, calcium, phosphor, and liver enzymes) was normal. Cranial CT was normal. Phenytoin (5 mg·kg^–1^·d^–1^) and total parenteral nutrition were started on November 28, 2009. Nasal and pharyngeal swab specimens obtained on November 26,2009 were positive for H1N1, based on real-time (RT)-PCR.

The patient’s fever did not recur since November 29, 2009 and she was extubated. Her neurologic examination after she was extubated showed that she had confusion, dysphasia, generalized hypotonia, diminished deep tendon reflexes, and no nuchal rigidity. ‘Electroencephalography (EEG) showed parieto-occipital alpha at 9-10 Hz and 4-5 Hz theta slow waves, which was compatible with mild encephalopathy’ Cranial magnetic resonance imaging (MR I) and cranial MR I angiography were normal.

Due to new-onset seizures, altered consciousness, immunocompromised state, and ongoing thrombocytopenia, we decided against lumber puncture, just after she was extubated [2]. Coagulation screening test results were normal; ANA, anti-DNA, and anticardiolipin antibodies were negative.

Cerebrospinal fluid was drawn on December 15, 2009 when her general condition was improved and chemotherapy was resumed, and had a protein value of 0.3 g L—1 (normal range: 0.15-0.5 g L—1) and glucose of 50 mg dL—1, without any cells in the cytospin slide. The patient’s pneumoniae recovered clinically and radiologically by December 15, 2009. Dysphasia and generalized hyponia resolved gradually by January 1, 2010. The patient’s follow-up EEG taken on February 3, 2010 was normal.

Phenytoin was stopped on February 3, 2010. After consolidation therapy (December 15-20,2009), the patient received allogeneic stem cell transplantation from a full-matched sibling (March 2, 2010). As of November 1, 2011, the patient had a Karnofsky score of 100% for 20 months without recurrence of neurologic deficit.

## DISCUSSION

A patient with acute neurological complications associated with H1N1 infection was reported to have laboratory- confirmed H1N1 infection of the respiratory tract and associated seizures, and encephalopathy or encephalitis within 5 d of the onset of influenza-like symptoms, without evidence of an alternative etiology. Encephalopathy was defined as altered mental status lasting >24 h [3,4] and encephalitis was defined as encephalopathy plus ≥2 of the following: fever >38 °C, focal neurological signs, CSF pleocytosis, EEG findings indicative of encephalitis, and abnormal neuroimaging indicative of infection or inflammation [3]. As such, the presented neutropenic patient fulfilled the criteria of encephalitis associated with flu-like symptoms, which were attributed to H1N1 superimposed with bacteremia due to Klebsiella pneumoniae.

Recovery of encephalopathy despite withdrawal of imipenem eliminates the diagnosis of imipenem-associated encephalopathy [4,5]. To the best of our knowledge the literature contains no evidence of a higher incidence of neuropsychiatric adverse events (NPAEs) in patients treated with oseltamivir—an antiviral used to treat influenza— than in those that receive a placebo or no antiviral treatment; therefore, we think that encephalitis in the presented patient was likely to have been caused by H1N1, which is consistent with reports stating that the risk of NPAEs in influenza patients is significantly higher than in the general population, [6,7]. [3] although the incidence of H1N1-associated encephalopathy is as low as 0.89% (3 out of 336 cases) [3] and 7% [4].

In our review of 25 cases of H1N1-associated encephalopathy/ encephalitis, only 3 (12%) were adults while the rest (88%) were children. None of the reported cases had an underlying disease, and the time from the onset of respiratory symptoms to the onset of neurological symptoms was 1-4 d.[8,9,10,11,12,13,14,15,16,17,18,19,20,21,22].

In our literature review, CSF findings in the reported cases were unremarkable, except pleocytosis with/without mild elevation in the protein level in seven cases with H1N1-associated encephalopathy/encephalitis out of 25 [8,12,14,15,16,20,21] and we found only one case with elevated CSF pressure [8]. In each case, though, the H1N1 virus was isolated from nasopharyngeal swab specimens via enzyme immunoassay and or PCR, none of the CSF specimens yielded H1N1 virus, except for Sanchez-Torrent’s [17] case in which lumbar puncture was traumatic.

All patients received oseltamivir with/without acyclovir or antibiotics.

The 2 of the reported cases whose EEG findings were consistent with encephalopathy were on the first day of oseltamivir when neurological complications developed. [4,12].Two adults progressed to encephalitis lethargica [8,14] and 4 children to acute necrotizing encephalopathy, [16,19,21,22] while other reported cases presented with encephalomyelitis,[10] meningoencephalitis,[9] or pseudobulbar palsy,[11] with accompaniment of severe intracranial hypertension.[18]

While CT or MR I findings were normal in patients that recovered quickly (2-7 d),[12,13,15,17,20] those with a complicated course [8,10,14,16,18,19,21] had radiological abnormalities, including unilateral or bilateral hyperintense signals in T2-weighted MR I images of thalamus, perirolandic area, basal nuclei, cerebellum, brainstem, medulla and cervical cord with [16,18] without [8,9,10,11,14,19,21] findings of increased intracranial pressure. The MR I findings were observed to have completely or slightly reversed in follow-up images.[11,21]

These reversible lesions were attributed to the transient development of intramyelinic edema, resulting in a transient decrease in the diffusion of the lesions—a common finding in cases with radiological abnormalities.[11]

In our literature review full recovery was attained in 72% of patients, [4,9,10,11,12,13,15,17,18,20] while 16,6% required assisted ventilation,[10,13,18] as did the presented patient. Few patients had extrapyramidal and/or pyramidal sequelae due to the progression to encephalitis lethargica [8,14] (8%) and acute necrotizing encephalopathy (8%),[16,21] or following severe encephalopathy (4%). [10] Two patients with acute necrotizing encephalopathy were reported to progress to brain death. [19,22]

The differences between the presented patient and those previously reported are that the presented case was immunodeficient, her neurological findings resolved gradually over a longer period of time (30 d vs. 2-7 d) than those with a benign course and no radiological findings, which may have been due to hypoxia following cardiopulmonary arrest, and that encephalitis did not pursue a complicated course like in those with encephalitis lethargica or ANE, even though she was immunodeficient.

Mutation in the gene Ran-binding 2 (RANBP2) have been shown to be associated with familial or recurrent influenza-associated acute encephalopathy/encephalitis [23]. Hence there are reported cases with familial [20] or recurrent [11] H1N1-associated encephalopathy/encephalitis.

Proinflammatory cytokines play a role in the etiology of H1N1-associated encephalopathy/encephalitis.via inducing vascular endothelial injury and increasing blood-brain barrier (BBB) permeability, which enables them to penetrate into the central nervous system (CNS) through a damaged BBB, induce apoptosis of neurons and glia, and activate elevated glial release of cytokines, thereby inducing brain edema and damage [19,24] Cytokine storm may also contribute to apoptosis of liver cells, metabolic disorders, and coagulopathy [16]. In the presented patient the underlying febrile neutropenia may have contributed to the development of encephalopathy via an increase in cytokines. In contrast, the absence of a significant inflammatory response in CSF or lesion enhancement on MR I, [25] and the absence of elevated cytokines in CSF [11] raises questions about the primary role of cytokine hypersecretion in the etiology of H1N1-associated encephalopathy/encephalitis.

Although the H1N1 virus proliferates in microglia and astrocytes, inducing apoptosis, and cytopathic and proinflammatory cytokine production in vitro, [24] viral antigen or viral nucleic acid have rarely been detected in CSF or neural tissue, [17,25] suggesting that influenza rarely produces true neuroinvasive disease [26]. Systemic vascular abnormalities [18] and cerebral arteritis [27] were also suggested to play a role in the etiology of H1N1-associated encephalopathy/encephalitis. Yet, the underlying mechanisms of influenza-associated neurological illness remains unclear [25]. In the presented patient CT, MR I, and MR I angiography findings ruled out bleeding or thromboemboli. Progressive recovery of her mental status without any intrathecal chemotherapy, and normal CSF findings 15 d later during chemotherapy ruled out CNS leukemia as well. Pulmonary infection in the presented patient may have been aggravated by the superimposition of H1N1 to Klebsiella pneumoniae, as previously reported [28].

In conclusion, the presented case indicates that during pandemics of influenza-like diseases, epidemic viruses like H1N1 should always be a consideration in the etiology of seizures in leukemic patients with febrile neutropenia, and that early initiation of treatment of H1N1 infection has the potential to reduce the associated morbidity and mortality rates.

## CONFLICT OF INTEREST STATEMENT

The authors of this paper have no conflicts of interest,including specific financial interests, relationships, and/or affiliations relevant to the subject matter or material sincluded.
